# The Accuracy of On-Call CT Reporting in Teleradiology Networks in Comparison to In-House Reporting

**DOI:** 10.3390/healthcare9040405

**Published:** 2021-04-01

**Authors:** Svea Storjohann, Michael Kirsch, Britta Rosenberg, Christian Rosenberg, Sandra Lange, Annika Syperek, Frank Philipp Schweikhard, Norbert Hosten

**Affiliations:** Department of Radiology, Universitätsmedizin Greifswald, 17475 Greifswald, Germany; Michael.Kirsch@med.uni-greifswald.de (M.K.); britta.rosenberg1@uni-greifswald.de (B.R.); Christian.Rosenberg@jsd.de (C.R.); sandra.lange@uni-greifswald.de (S.L.); annika.syperek@med.uni-greifswald.de (A.S.); ps143182@uni-greifswald.de (F.P.S.); norbert.hosten@med.uni-greifswald.de (N.H.)

**Keywords:** telemedicine, reporting, quality control, resident, diagnostic error

## Abstract

(1) Background: We aimed to compare the accuracy of after-hours CT reports created in a traditional in-house setting versus a teleradiology setting by assessing the discrepancy rates between preliminary and final reports. (2) Methods: We conducted a prospective study to determine the number and severity of discrepancies between preliminary and final reports for 7761 consecutive after-hours CT scans collected over a 21-month period. CT exams were performed during on-call hours and were proofread by an attending the next day. Discrepancies between preliminary and gold-standard reports were evaluated by two senior attending radiologists, and differences in rates were assessed for statistical significance. (3) Results: A total of 7209 reports were included in the analysis. Discrepancies occurred in 1215/7209 cases (17%). Among these, 433/7209 reports (6%) showed clinically important differences between the preliminary and final reports. A total of 335/5509 of them were in-house reports (6.1%), and 98/1700 were teleradiology reports (5.8%). The relative frequencies of report changes were not significantly higher in teleradiology. (4) Conclusions: The accuracy of teleradiology reports was not inferior to that of in-house reports, with very similar clinically important differences rates found in both reporting situations.

## 1. Introduction

With the rise of teleradiology, it has become possible to physically separate the sites of image acquisition and interpretation of the resulting scans. Today, radiology reports are not necessarily created at the same facility in which the images are acquired; instead, scans may be read and reported on remotely by physicians in teleradiology networks. Teleradiology networks typically consist of institutions providing 24/7 readings of imaging studies and corresponding requesting institutions, such as smaller hospitals that do not have the financial or personnel means to ensure the around-the-clock presence of radiologists in their imaging departments [1]. The European Society of Radiology (ESR) conducted a survey to obtain the current status of teleradiology [2]. In total, 70.8% out of 25 National societies that responded to the survey answered that in their country, the outsourcing of worklists to teleradiology companies is practiced, i.e., without direct contact between the radiologist and the patient.

In comparison to in-house reporting, “teleradiologists typically do not have access to additional information, including prior studies, plain films, or clinical data, which may assist in-house radiologists in image interpretation” (quoted verbatim from [3]; also [4]). In the teleradiology setting, the reader has to rely on the often-scarce information provided by the referring physician. To protect medical data, prior films and medical files cannot always be accessed remotely when reporting by teleradiology. Direct communication between the radiologist and the patient, which is considered a valuable source of clinical information [5], is rarely possible in this setting. Even if it is not always feasible in the daily routine of in-house diagnostics, it represents another source of information that is lost in teleradiology.

According to German law, teleradiology is intended as an exception to close gaps in care. It is authorized for reporting at night, on weekends, and on bank holidays (24/7 teleradiology as another exception may be approved upon request under certain conditions that must be met). Another requirement based on quality assurance (QA) aspects in the German teleradiology setting is the so-called “regional principle”. According to this, the teleradiologist may only work for locations that can be reached within a period of time necessary for emergency care (approx. 45–60 min). In addition, there are strict requirements for the professional experience and qualifications of the radiologists participating in teleradiological reporting [6,7,8,9,10].

A considerable number of existing quality control studies have been conducted in North America. They identified a variety of items which might influence the quality of after-hours reporting. Possible influencing factors were evaluated, such as whether reports were done on a weekend versus a week day, whether reports were done during the hours of a shift or not, and the complexity of a case [11]. There are some studies that reported statistics of a QA program tracking reported disagreements that occurred in observing CT examinations [3,12,13]. In these studies, residents were not involved in the reporting. To the authors’ knowledge, no work comparable to the available studies has been reported from Germany to date.

As such, this study was conducted to evaluate the relationship between the imaging setting (teleradiology/network reporting vs. in-house reporting) and the frequency of discrepancies between teleradiology and in-house reports. We evaluated the distribution of neuroradiological examinations, as these are often evaluated separately in quality control studies. We hypothesized that teleradiology reporting would produce more discrepancies—caused, for example, by the lack of contact between the radiologist and the patient, possibly missing preliminary examinations or insufficient clinical information.

## 2. Materials and Methods

The present study was conducted prospectively. It was reviewed and approved by the local ethics committee and the staff council representing the affected doctors. The teleradiology operation was approved by the local authorities in 2014. In accordance with national laws and regulations, all participating radiologists were informed of the use of their reports in the study. Consent for the necessary diagnostic measure was obtained from all patients involved in the study as far as they were able to give their consent. There was no additional or special risk for the patients from the study. All patient data in the reports were anonymized for evaluation in consideration of the relevant data protection regulations.

CT imaging was chosen as the imaging modality of study since it represents the most frequently requested imaging modality outside core working hours, for the interpretation of which the radiologist is in demand.

### 2.1. Reporting Process during On-Call Shifts

During nighttime hours (10 p.m. to 7 a.m.) and during the daytime hours on weekends and bank holidays (7 a.m. to 10 p.m.), in accordance with the German teleradiology law rules, on-call radiologists created preliminary reports for CT studies that were either acquired in-house, on our own scanners, or received via the teleradiology network (8 smaller hospitals). The files were sent with point-to-point encryption via a virtual private network (VPN). As is common practice in radiology departments, the on-call radiologist was able to involve an attending radiologist if they decided that the case required a higher level of expertise (for details on the roles of the different readers, see Table 1). During the next regular daytime shift, all of these reports were reviewed by an attending radiologist and corrected if necessary. The resulting proofread final reports were considered to be gold-standard. A correlation of the gold-standard findings with the clinical outcome of patients was not possible, as all data including the patient data and reporting radiologist were required to be deleted in accordance with data protection regulations.

### 2.2. Availability of Supplementary Information

With in-house imaging, radiologists had full access to all information available on the patient within the Picture Archiving and Communication System (PACS), as well as the hospital and radiological information systems (RIS). This includes prior studies and clinical data such as secondary diagnoses and operative reports. Further information could be acquired by communicating with the referring physician and patients themselves.

For reporting in the teleradiology network, the on-call radiologist could communicate with the referring physician on site and, more importantly, communicate with the technician on site performing the exam, usually focusing on the proposed examination protocol. There was no direct patient–radiologist communication. The written request from the referring colleague communicated clinical information. Prior studies could not be accessed since the requesting and receiving hospitals did not share a PACS or RIS.

### 2.3. Exclusion Criteria

CT studies which fulfilled one or more of the following criteria were excluded: scans that were not reported the next weekday; scans where the initial report was edited before the next weekday (the initial findings were then overwritten and could no longer be reviewed; any changes made to the report could no longer be traced); scans aborted mid-examination; scans related to an intervention, report created by attending; no verification (in this case, the preliminary report could not be released and a comparison with the gold-standard was impossible at the time of the study).

The contact to an attending was not seen as an exclusion criterion, as it is common practice in both in-house reporting and teleradiology.

### 2.4. Data Processing

The preliminary on-call reports and the proofread versions were retrieved from our PACS and anonymized by a member of the study group. All data containing the identity of the patient, the reporting radiologist, or the hospital in which the scans were acquired were deleted.

The blinded reports were compiled side-by-side into a single document in order to allow for direct comparisons. In order to evaluate the report quality, both versions (on-call and proofread by a senior attending) were compared, and any apparent differences were highlighted.

### 2.5. Assessing the Discrepancy Level

If any discrepancies between the on-call report and the proofread final report were identified, the compiled documents were presented to two senior radiologists (>20 years work experience each), who assessed the changes in terms of their clinical and therapeutic consequences. The two readers made their decisions independently. Discrepancies were assigned to five severity levels and subsequently categorized to groups already used in previous publications in the context of studies on second-opinion consultations in radiology (see Table 2) [14,15] (Score 2: addition of a secondary diagnosis such as “maxillary sinus mucocele” when asked about acute ischemia; Score 3: clinically unimportant change in interpretation such as “radiopaque foreign material” to “DD clips”; Score 4: e.g., addition of a missed fracture; Score 5: clinically important change in interpretation such as the age of an ischemic infarction). In case of disagreement, the two readers would discuss this and reach a final consensual decision. One of the readers was also involved in the finalization of on-call reports. There was an interval of several months between the two activities so that no recollection of the circumstances of individual examinations or findings could be assumed.

### 2.6. Statistical Analysis

#### 2.6.1. Sample Size

Our aim was to minimize changes in the reporting patterns which might occur if radiologists were aware of an ongoing monitoring process. This is why, instead of determining a certain case number, we instead assigned a period (21 months) over the course of which all CT reports would be evaluated. As a result, because a study duration was assigned rather than a required number of cases, there was a larger number of cases than a pure power calculation would warrant. This was done with the aim of minimizing the on-call radiologists’ required attention over time.

#### 2.6.2. Testing

We calculated the absolute and relative frequencies of different severities of report changes and considered the acquisition locations as a risk factor for report changes. Statistical significance of differences in the examined frequencies of discrepancies between comparison groups was tested using the chi-square test. In addition, the chi-square test was used to evaluate the distribution of neuroradiological cases. This aimed at making comparison with other studies easier: Neuroradiological examinations are often evaluated separately in quality control studies. In this study, we intended to investigate emergency imaging of all body regions. Statistical analysis was performed using SPSS for Mac OS (Version 25; IBM, Chicago, IL, USA).

## 3. Results

### 3.1. Number of Cases

**Figure 1:**
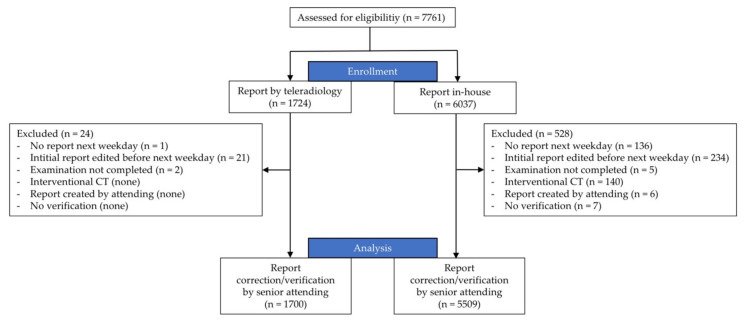
Flowchart of study. During the study period, 7761 consecutive after-hours CT scans were performed. After applying the exclusion criteria, a total of 7209 reports were included in the study.

Within the planned study period, a total of 6037 in-house CT reports and 1724 teleradiology reports were requested outside our hospital’s core working hours (nighttime hours: 10 p.m. to 7 a.m.; weekends and bank holidays: 7 a.m. to 10 p.m.) (Figure 1). For 136/6037 (2.3%) and 1/1724 (0.1%) cases, no digital report was created, 234/6037 (3.9%) and 21/1724 (1.2%) reports were edited under unclear circumstances, 5/6037 (0.1%) and 2/1724 (0.1%) scans were aborted mid-procedure, 140/6037 (2.3%) scans were directly related to an intervention (performed by an attending), 6/6037 (0.1%) reports were created by an attending, and in 7/6037 (0.1%) cases, no “gold-standard” report was available at the end of the study period. After excluding these cases, 5509 in-house reports and 1700 teleradiology reports remained for analysis. There were 24 cases excluded from the teleradiology arm (1.4%) and 528 from the in-house arm (8.7%). The higher percentage of in-house cases that were excluded had several reasons: interventional CT, whose reports were excluded because it is performed by attendings, was only performed in-house (without intervention 388 cases were excluded, 6.6%). Immediate clinical feedback led to more reports being changed in-house during the night. Teleradiology reports were reported without additional consultation and therefore more promptly delivered. Unlike in-house reports, they had to be reported; the report could not be delayed till the next morning, e.g., in agreement with the referring physician.

### 3.2. Frequency of Report Changes in In-House/Teleradiology Reporting

To investigate the influence of the examination setting on report discrepancies, we calculated error rates and risks in both groups. In the 7209 CT reports which were included in the analysis, discrepancies occurred in 1215 cases (16.9%). A total of 433 clinically important differences between the preliminary report and gold-standard report were identified (6%) (see Table 2).

**Figure 2:**
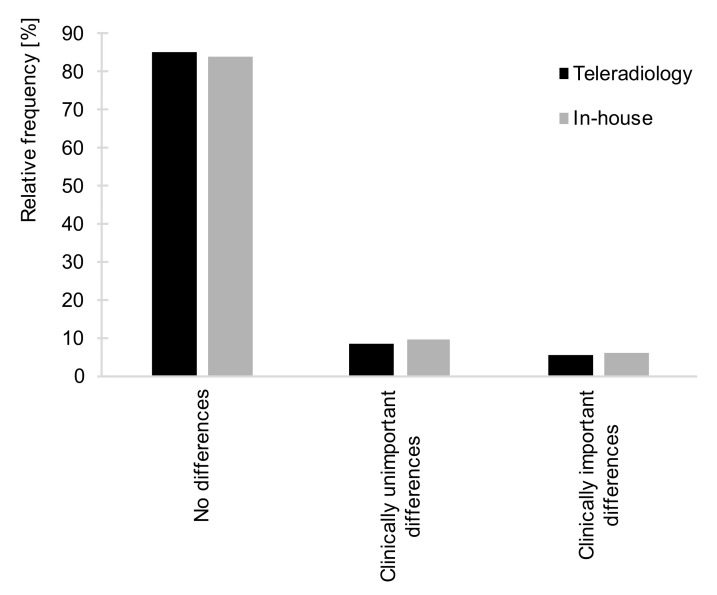
Relative frequencies of no differences, clinically unimportant differences, and clinically important differences for the teleradiology (black bars) versus in-house setting (grey bars). The relative frequencies of reports to which no changes were made; clinically unimportant and clinically important differences did not differ significantly between the teleradiology and the in-house setting (*X^2^*(2) = 1.828, *p* = 0.401, *n* = 7209). Found in 5.8% vs. 6.1% of cases, respectively, clinically important differences to CT reports were similarly rare in both teleradiology reporting and in-house.

In the in-house setting, clinically important differences occurred in 335 of 5509 reports (6.1%). Among the 1700 teleradiology reports that were included, 98 underwent clinically important differences (5.8%) (see Figure 2, Table 2).

Overall, the frequency of any kind of report changes was neither significantly higher nor lower for the teleradiology reports compared to in-house imaging (*p* > 0.05). This suggests that in-house reporting and reporting of CT exams transmitted via teleradiology did not differ significantly with regard to reporting errors.

### 3.3. Scanned Body Regions

**Figure 3:**
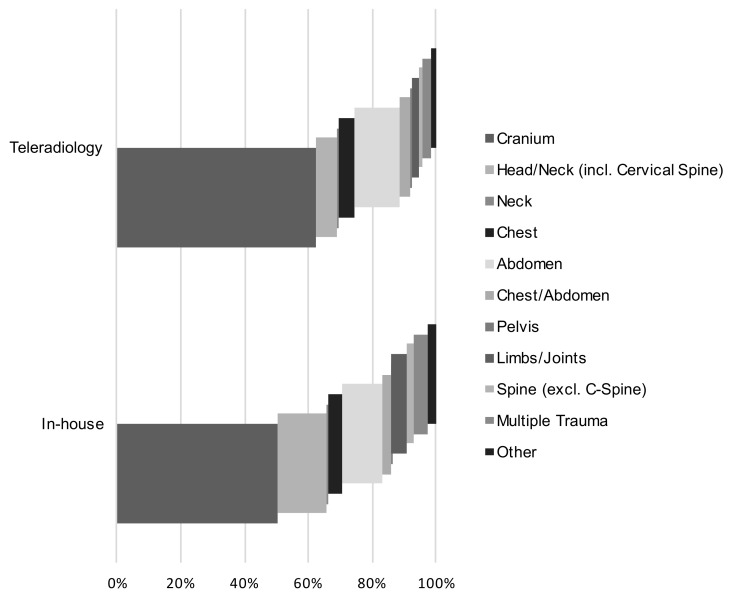
Proportion of examined body regions in the total number of examinations for both settings. A total of 83.8% (teleradiology) and 78.4% (in-house) of all examinations consisted of a cranial CT, a head and neck CT or an abdominal CT. The portions were comparable in both study arms.

To exclude the possible influence of different compositions of the CT reports evaluated in teleradiology and in-house studies, we compared the anatomical regions examined in each arm (see Table 3 for details on the different types of examination). For both in-house imaging and teleradiology, cranial CTs were the most frequently requested examinations, followed by head/neck and abdominal studies. The absolute number of CT images of each body region and their relative frequency in relation to the total number of CT studies in the respective setting type are provided in Table 3 and Figure 3. Results suggest the two types of reporting (network/teleradiology vs. in-house) did not differ in terms of the composition of the exam types.

### 3.4. Distribution of Reader Groups

Reports were created by 20 different radiologists. All radiologists were equally involved in both in-house and teleradiology reporting. Residents created 5005/7209 (69%) reports. The remaining 2204/7209 cases (31%) were read by board-certified radiologists. The distributions did not differ significantly between in-house and teleradiology reports (residents 69% vs. 71%; board-certified radiologists 31% vs. 29%).

This suggests that the work experience of the reporting radiologists did not differ significantly between in-house reporting and teleradiology.

## 4. Discussion

In the present study, report changes did not occur more frequently in the teleradiology setting than in the in-house comparison group. Teleradiology provides affordable full-time access to diagnostic imaging for smaller hospitals [16] by capitalizing on the 24/7 presence of radiologists in larger hospitals. Thrall [17] pointed out that emergency teleradiology has a limited range of indications and does not need results of prior examinations or clinical history; it therefore works well. Nevertheless, adequate report quality should be a top priority in teleradiology: today’s teleradiology reporting of emergencies may extend into daytime network reporting [1] and become the new standard. In-house and teleradiology reports did not differ with regard to reporting errors (Figure 2). Clinically important differences to the preliminary reports were made in 6.1% (in-house, n = 335) and 5.8% (teleradiology, n = 98) of cases, respectively.

### 4.1. Frequency of Report Changes in In-House and Teleradiology Reports

The accuracy of reports generated by teleradiologists is a recurrent concern. According to the authors’ knowledge, there is a lack of published QA data from German teleradiology networks. Due to the special legal regulations in Germany, comparability with international studies is limited. Additionally, the QA studies available for teleradiology were conducted without the participation of residents.

The clinically important difference rates observed in this study’s teleradiology arm are similar to the 2010 findings by Platt-Mills et al. [3]. Their study, which included head and body CT, revealed that major changes occurred in 6% of reports, while 73% remained entirely unchanged. Teleradiologists there also did not have access to any preliminary images. A study by Hohmann et al. [12] also reported 79% examinations without discrepancies. Previous examinations were provided to the teleradiologists. For both of these studies, teleradiology reports were audited at the department in which the images were acquired rather than at the teleradiology facility itself.

In a 2003 publication by Erly et al. [13], only emergency cranial CT reports were examined. Major discrepancies were found to be less common. In total, 2.0% of the reports created by board-certified general radiologists via teleradiology were subject to significant disagreement. Complete agreement was observed in 95% of cases. However, the examinations were sent as an image file. In this way, only the brightness and contrast of the images could be edited by the radiologists.

### 4.2. Frequency of Report Changes Depending on Other Factors

Several studies have found that in the context of in-house reporting, the discrepancy rate correlates *inversely* with work experience [18,19,20,21]. Meanwhile, Cooper et al. [22] and Mellnick et al. [23] propose that a *positive* correlation between work experience and report discrepancies stems from the increasing responsibility that comes with increased work experience [23]. They found that the risk for report changes was significantly higher when the reader had less than four years of work experience. Lam et al. [24] found that discrepancies were much more likely to occur during the night shift. Developing a protocol for communicating discrepancies between on-call and final reports is essential. The most dreaded consequence of a discrepancy—a change in patient outcome—rarely occurs and only takes place in less than one percent of cases [19,25] but may be necessary and must be addressed. In our institution, difficult cases which gave rise to discrepancies (such as appendicitis, urinary calculus, small-bowel obstruction, diverticulitis) [25] are discussed in the daily morning rounds. Residents may thus familiarize themselves with typical off-hour problems before they start taking calls.

### 4.3. Does a Lack of Clinical Information and Access to Prior Studies Affect Report Quality?

If it is too costly and time-consuming for the teleradiologist to obtain clinical information, there is a risk that examinations will be interpreted with incomplete preliminary information [17]. So far, there are few data on whether a relative lack of clinical information affects the quality of teleradiology reports. Millet et al. [26] found that the absence of clinical information did not negatively influence diagnostic accuracy in abdominal CT. Mullins et al. [27] saw reports for stroke CTs improve when clinical data were available; MR results did not change, however. A review by Loy and Irwig [28] cited several papers focused on the bias inherent to clinical information, which may inadvertently direct the radiologist’s attention toward evidence of the clinically suspected diagnosis. Interestingly, in light of this, sufficient clinical information was found to help to establish a rational examination protocol in a study by Dang et al. [29].

The limitations of this study result from the strict requirements regarding the anonymization of the collected data. It was not possible to calculate the influence of individual radiologists on the group performance. In addition, it was not possible to follow up on patients whose examination underwent a change. Thus, only the final report could be used as a gold standard. The influence of changes on the outcome of patients could not be determined.

## 5. Conclusions

In conclusion, teleradiologists need to work with the lack of personal contact with patients, technical staff, and referring physicians. This did not compromise the accuracy of CT reports compared to a traditional in-house setting. The frequency of reports to which changes were made did not differ significantly between the teleradiology and the in-house setting. Clinically important differences to CT reports were similarly rare in both settings. Our study, as such, establishes teleradiology as a realizable way of providing after-hours radiology services.

## Figures and Tables

**Table 1:** Role of the different members of the Department of Radiology involved in the present study. The upper and middle boxes refer to reporting, while the lower box refers to the acquisition of the study data used for assessing discrepancies.

Radiologist on call	First-line reporting.
Attending	Could be consulted by the radiologist taking call; proofread reports the next morning/workday; the resulting final report was considered “gold-standard” for this study.
Senior radiologist	Two attendings specializing in radiology and neuroradiology, respectively; independently, they graded differences as either “clinically unimportant” or “clinically important” and differences as either “in detection” or “in interpretation”.

**Table 2:** Consensus score [14,15] of final interpretation versus preliminary interpretation in in-house and teleradiology reports. In-house and teleradiology reports were subject to clinically unimportant and clinically important differences at similar rates.

Discrepancies		Setting		
		In-House [n (%)]	Teleradiology [n (%)]	Sum [n (%)]
1	No difference	4633 (84.1)	1452 (85.4)	6085 (84.4)
2	Clinically unimportant difference in detection	168 (3)	31 (1.8)	199 (2.8)
3	Clinically unimportant difference in interpretation	373 (6.8)	119 (7)	492 (6.8)
4	Clinically important difference in detection	193 (3.5)	51 (3)	244 (3.4)
5	Clinically important difference in interpretation	142 (2.6)	47 (2.8)	189 (2.6)
sum		5509 (100)	1700 (100)	7209 (100)

**Table 3:** Type of CT examinations included in this study for both settings. The three most frequently examined body regions are highlighted. The proportion of exams from the neuroradiological field, which is often evaluated separately in quality control studies, did not differ between the two groups, as indicated by a low effect size, *Cramers V*. However, there was a statistical difference due to the large number of cases included. (65.4% vs. 69.2%; *X^2^*(1) = 8.127, *p* = 0.004, *n* = 7209, *Cramers V* = 0.034).

Scanned Region	Teleradiology		In-House	
	n	[%]	n	[%]
Cranium	1057	62.2	2777	50.4
Head/Neck (incl. Cervical Spine)	119	7.0	828	15.0
Neck	4	0.2	35	0.6
Chest	83	4.9	239	4.3
Abdomen	248	14.6	714	13.0
Chest/Abdomen	48	2.8	124	2.3
Pelvis	11	0.6	23	0.4
Limbs/Joints	35	2.1	266	4.8
Spine (excl. C-Spine)	24	1.4	108	2.0
Multiple Trauma	41	2.4	241	4.4
Other	30	1.8	154	2.8
sum	1700	100.0	5509	100.0

## Data Availability

Not applicable.
